# Virtual embolization for treatment support of intracranial AVMs using an interactive desktop and VR application

**DOI:** 10.1007/s11548-021-02532-9

**Published:** 2021-11-22

**Authors:** Ulrike Sprengel, Patrick Saalfeld, Janneck Stahl, Sarah Mittenentzwei, Moritz Drittel, Benjamin Behrendt, Naoki Kaneko, Daniel Behme, Philipp Berg, Bernhard Preim, Sylvia Saalfeld

**Affiliations:** 1 Department of Simulation and Graphics, Otto-von-Guericke University Magdeburg, Universitätsplatz 2, 39106 Magdeburg, Germany; 2 Department of Radiological Sciences, David Geffen School of Medicine, University of California, Los Angeles, USA; 3 Department of Neuroradiology, University Hospital Magdeburg, Magdeburg, Germany; 4 Department of Fluid Dynamics and Technical Flows, Otto-von-Guericke University Magdeburg, Forschungscampus STIMULATE, Magdeburg, Germany; 5 Department of Simulation and Graphics, Otto-von-Guericke University Magdeburg, Forschungscampus STIMULATE, Magdeburg, Germany

**Keywords:** AVM, VR, Embolization

## Abstract

**Purpose:**

The treatment of intracranial arteriovenous malformations (AVM) is challenging due to their complex anatomy. For this vessel pathology, arteries are directly linked to veins without a capillary bed in between. For endovascular treatment, embolization is carried out, where the arteries that supply the AVM are consecutively blocked. A virtual embolization could support the medical expert in treatment planning.

**Method:**

We designed and implemented an immersive VR application that allows the visualization of the simulated blood flow by displaying millions of particles. Furthermore, the user can interactively block or unblock arteries that supply the AVM and analyze the altered blood flow based on pre-computed simulations.

**Results:**

In a pilot study, the application was successfully adapted to three patient-specific cases. We performed a qualitative evaluation with two experienced neuroradiologist who regularly conduct AVM embolizations. The feature of virtually blocking or unblocking feeders was rated highly beneficial, and a desire for the inclusion of quantitative information was formulated.

**Conclusion:**

The presented application allows for virtual embolization and interactive blood flow visualization in an immersive virtual reality environment. It could serve as useful addition for treatment planning and education in clinical practice, supporting the understanding of AVM topology as well as understanding the influence of the AVM’s feeding arteries.

**Supplementary Information:**

The online version supplementary material available at 10.1007/s11548-021-02532-9.

## Introduction

Arteriovenous malformations (AVMs) are pathologic vessel malformations describing a tangle, the so-called *nidus*, of aberrant arteries linking the blood feeding arteries directly to the veins without containing a capillary bed. The connecting vessels between the nidus and the parenting artery are called *feeders* and are of particular interest for treatment since they supply the AVM. Cerebral AVMs can rupture and/or cause a lack of oxygen in the surrounding area, which may lead to neurological disorders with fatal outcome [17]. AVM treatment is complex and challenging, depending on type and location, and often includes embolization, stereotactic radiosurgery, microsurgery or a combination thereof [4].

We describe an application to support patient-specific planning of cerebral AVM treatment focusing on embolization of the AVM’s feeder arteries. To embolize the feeders, a sclerosing agent is used in clinical practice. Since the embolization in one feeder alters the blood flow in the other feeders, which may increase their chance of rupture [21], the order of embolization is highly relevant. Our application visualizes the blood flow of different embolization combinations and steps, which enables the interventional neuroradiologist to better decide about the embolization strategy based on simulated blood flow. In addition to a desktop mode, we offer a virtual reality (VR) mode to allow an improved assessment of the spatially complex vascular structures. For neuroradiological interventions, virtual reality simulators have been developed, including haptic feedback [15]. Given the complex anatomy of the brain’s vasculature, VR has great potential to bridge the gap between 2D image analysis and the 3D clinical setting [10] and adds value for neurosurgery training [2, 13]. Ng et al. [14] presented a surgical planning tool for AVMs using a stereoscopic monitor and could show that it allowed to identify critical feeders and draining vessels. However, a representation of AVMs with an integrated visualization of real-time blood flow and interactive embolization strategies is not available yet.

**Fig. 1:**
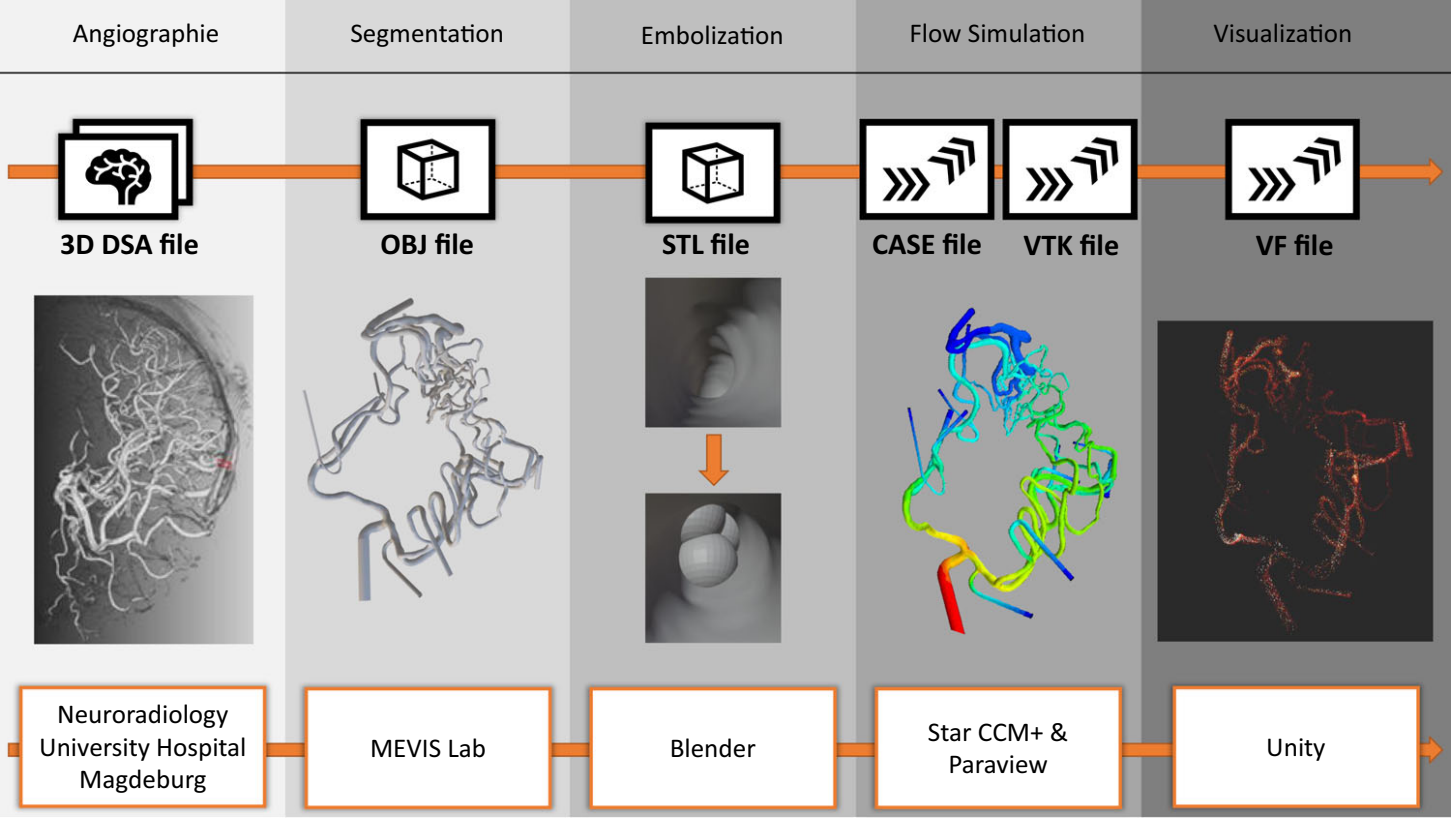
Workflow starting with the clinical 3D DSA, over the segmentation, simulated embolization, flow simulation and the 3D visualization. The visualization is realized for a desktop and a VR application

For a satisfying blood flow visualization, millions of particles are required. Our application comprises this amount of particles for an intuitive presentation of AVMs and their internal blood flow. In a pilot study, we tested our application which was successfully used for three AVM cases. We evaluated our approach with two clinical experts, who pointed out the advantages of a truly virtual treatment planning of an AVM embolization without harming the patient. With the presented application, a step-by-step embolization can be virtually conducted in order to avoid strong changes in pressure, which is crucial in AVM treatment.

## Materials and methods

In this section, we describe the AVM data and our application. Our application is based on and significantly extends earlier work [18]. We developed a new pipeline including the VR application and VR-based exploration techniques, a novel conversion from simulated blood flow data (i.e., from VTK to VF files), an improved visualization of embolization and blood flow and evaluated and segmented a new set of datasets from patient-specific AVM cases. Finally, an evaluation with two experienced neuroradiologist was conducted, discussing the embolization strategy in detail.

### Medical image datasets

**Fig. 2:**
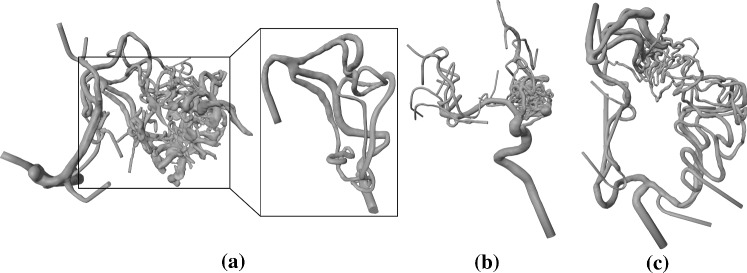
Segmented 3D surface models of the three AVMs: Case 1 with marked region of interest around the nidus (left) and cropped 3D model (right) (**a**), Case 2 (**b**) and Case 3 (**c**)

3D digital subtraction angiography (3D DSA) data of three patients were segmented and converted into 3D models. For Case 1, the 3D DSA data ($0.28 \times 0.28 \times 0.28\,\mathrm{mm}$) comprises a nidus, supplied by branches of the left posterior cerebral artery. By using a threshold-based segmentation (similar to the method described in [9]), 3D mesh models were extracted from the clinical image data. We created an example case containing only the feeders and a smaller section of the nidus (see Fig. 2a). This simplified representation of the AVM provides a clear introduction to the VR method, as the user can get to know different functionalities using a clear case.

To demonstrate the feasibility of the presented approach for patient-specific AVMs from clinical practice, complex segmentations without artificial reduction in the segmented 3D models were extracted, yielding Case 2 and Case 3 (see Fig. 2b, c). The patient of Case 2 suffers from a complex nidus located directly at the internal carotid artery. Furthermore, a flow-associated aneurysm is located at the feeder. 3D DSA image data was acquired with a resolution of $0.24 \times 0.24 \times 0.24\,\mathrm{mm}$. The AVM contained in Case 3 was acquired with 3D DSA ($0.47 \times 0.47 \times 0.47\,\mathrm{mm}$) as well. It exhibits feeders originating at the left middle cerebral artery and the left anterior cerebral artery. The draining veins are connecting directly into the superior sagittal sinus. The 3D models for Case 2 and 3 were extracted using thresholding as well.

### Virtual embolization and blood flow visualization

The goal of the virtual blood flow visualization is to support physicians in therapy planning of AVMs in an easy and comprehensible way. Due to the spatially complex anatomy of AVMs, we chose an immersive environment. For a realistic rendering, requiring millions of moving particles for blood flow visualization even in narrow vascular structures, we chose the VFX Graph from the cross-platform game engine Unity (Unity Technologies, Unity v2019.1.3f1, https://unity.com/). The processing of the data, which is required for the GPU-based VFX Graph, is shown in Fig. 1 and explained in the following.

#### Simulation of embolization

We simulated the embolization of a feeder artery by blocking this artery in the 3D model. The 3D model as well as all its altered variations is then used as input for a subsequent hemodynamic blood flow simulation, which will be described in the next section.

To achieve a realistic result, we reproduce the embolization as it is conducted in clinical practice [17]. In order to minimize perturbations in the hemodynamics and to reduce the blood supply to the veins, a sclerosant, i.e., a fine-grained embolic agent, is injected into the feeder arteries as close as possible to the nidus.

**Fig. 3:**
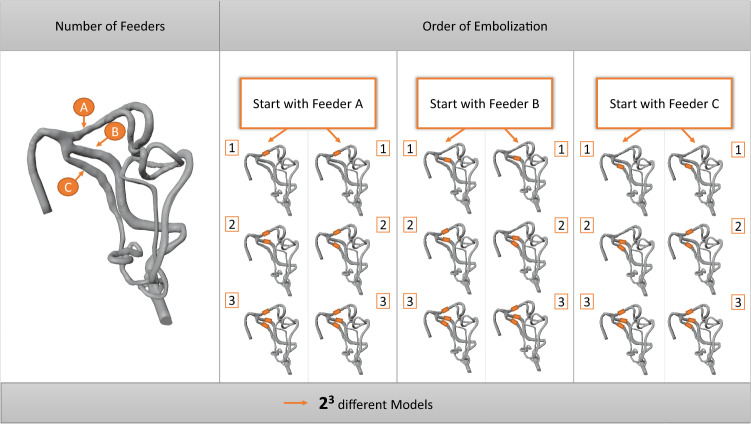
Illustration of the simulated embolization by blocking each feeder individually. For the example Case 1, this results in $2^3$ 3D models and subsequent simulations

To explore the influence of different embolization combinations of the arteries in order to find an optimal treatment strategy with respect to simulated blood pressure, we conducted simulations for each feeder blocked and unblocked, respectively. As a result, the simplified anatomy of Case 1 contains three feeders and therefore $2^3$ possible combinations of blocked or unblocked feeders yielding eight hemodynamic simulations (see Fig. 3).

**Fig. 4:**
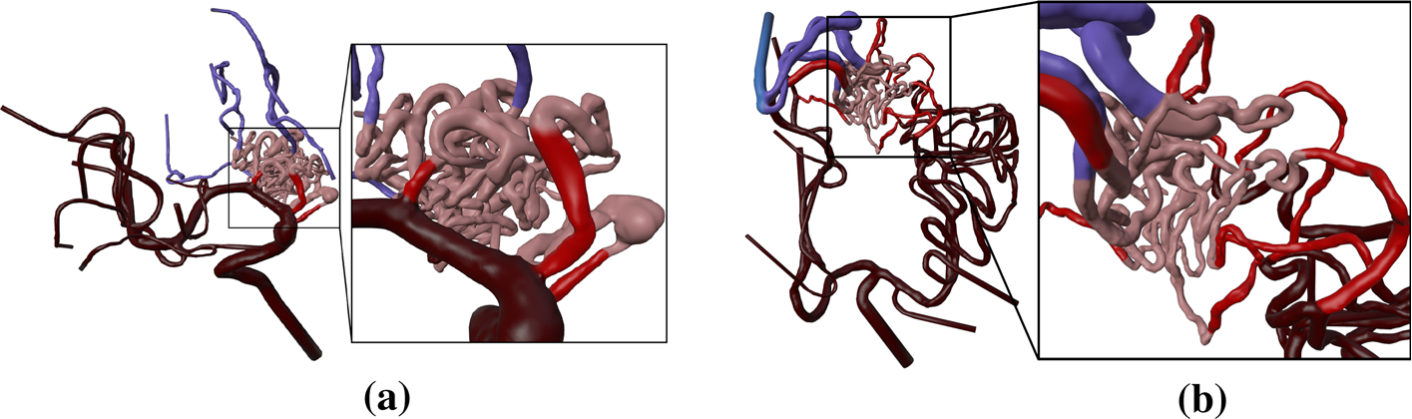
Colored 3D models of Case 2 (**a**) and Case 3 (**b**) with arteries in dark red, feeders in red, nidus in light red, draining veins in purple, veins in blue

We showcase the capabilities of our application with three cases (see Fig. 2). Case 1 represents a cropped part of a complex AVM and allows to give users an understanding of the functions and principle of the software; Case 2 and Case 3 are way more complex containing a tangled system of arteries. For a correct identification of feeders and a reliable simulation of an embolization, an expert interview with a physician was carried out. Case 2 has a more compact nidus where possible feeders can be identified more easily (see Fig. 4a). Three feeders are supplying the nidus, with the peculiarity of an aneurysm between the first feeder and the connected nidus. For Case 3, eight feeders were identified (see Fig. 4b). All of them are resulting from the branches of the left middle cerebral artery and the left anterior cerebral artery. For both cases, virtual embolization of the feeders was carried out by blocking the blood supply at the transition of the feeder into the nidus. These regions are visible by the color transition from red to light red (see Fig. 4).

#### Blood flow simulation

For each case and the altered 3D models (i.e., blocked vessel choices), we conducted a hemodynamic simulation to pre-calculate the blood flow with STAR-CCM+ 15.04 (Siemens PLM Software Inc., Plano, TX, USA). STAR-CCM+ is a computational fluid dynamics solver used for simulations in the industry as well as in research, offering many standard components for simulation. For the simulations carried out within this study, we followed the recommendations for hemodynamic modeling in intracranial aneurysms [5].

In detail, the 3D surface models were converted into volume meshes using polyhedral and prism cells with a base size of 0.15 mm for Case 1 and 0.2 mm for Case 2 and 3, respectively. Hence, three prism layers were generated at the luminal surface to resolve the velocity gradients. For inflow modeling, a constant inflow velocity of $0.1\,\frac{\hbox {m}}{\hbox {s}^2}$ (Case 1) and $0.39\,\frac{\hbox {m}}{\hbox {s}^2}$ (Case 2 and 3) was employed based on 7T phase-contrast MRI measurements that were acquired in the Circle of Willis of a healthy volunteer [6]. To account for the differences in vessel size between the representative volunteer and the Cases 1 to 3, the flow values were scaled according to the ratio of the cross-sectional inflow areas. We used zero-pressure boundary conditions at all outlets and assumed rigid vessel walls since no further information was available. Blood was modeled as incompressible ($\rho = 1055\,\frac{\hbox {kg}}{\hbox {m}^3}$) and Newtonian ($\eta = 4\,\mathrm{mPa} \cdot \mathrm{s}$) fluid and a laminar flow type was chosen. The simulated data were saved as binary-encoded EnSight Gold files.

#### Postprocessing for unity’s VFX graph

To use the VFX Graph, a vector field (VF) file representing a 3D texture is needed. First, the EnSight Gold files were converted to ASCII-coded VTK files using ParaView [1](Kitware Inc., New York, USA). Next, the VTK files were converted to VF files with a custom script, including a mapping of the data points to a Cartesian grid. The structure of VF files was created according to Unity’s (https://github.com/peeweek/VectorFieldFile). The level of detail can be adjusted with the step size of the grid during the conversion. For the examples presented in this work, we used a step size of 0.2 mm.

## Results

### Interactive blood flow application

**Fig. 5:**
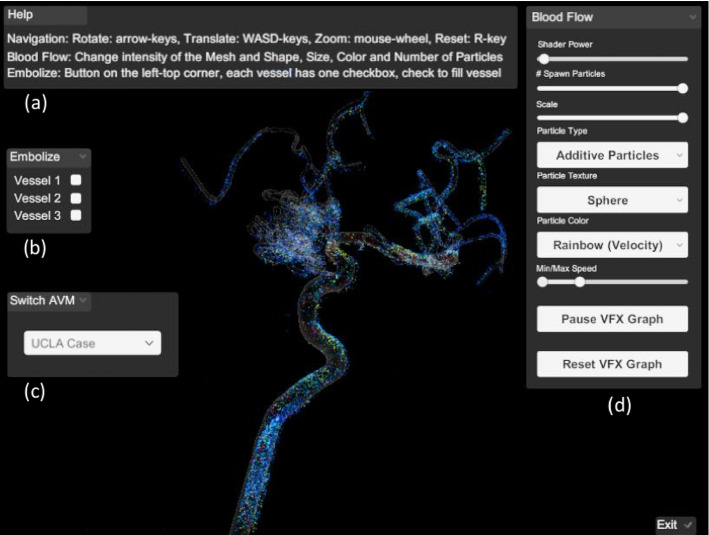
GUI of the application showing Case 2. The menu consists of **a** getting help for the interaction, **b** the possibility to embolize feeders, **c** a dropdown menu for switching between cases, **d** a sub-menu to edit the parameters regarding the blood flow and the shading of the model itself

The GUI of our immersive AVM planner is depicted in Fig. 5. The user can explore the 3D AVM model by rotating, translating and scaling it with the keyboard. The user interface enables the user to switch between different datasets and to each of the dataset’s variations, and alter the blood flow and opacity. A ghosted view technique was used to show underlying particles in the 3D model [3]. The ghosted view is a rendering technique to tackle occlusion problems. The transparency of the surface is based on the viewing angle, thus the user can see the blood flow inside the vessels without loosing the anatomical context of the vessel walls [8].

The user can alter properties of the underlying VFX Graph including its four components: spawn, initialize, update and output. The number of spawned particles, the number of simultaneously existing particles, the lifetime of the particles and their size are adjustable.

**Fig. 6:**
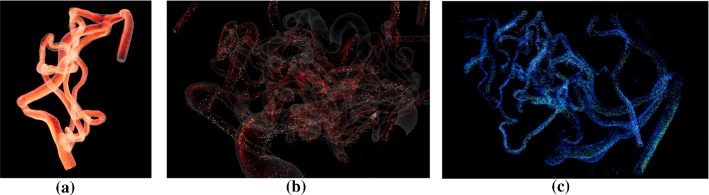
Example for the three cases using different color scales: **a** Case 1 with additive blending **b** Nidus of case 2 using the heated body map color scale **c** Nidus of case 3 with the rainbow color scale

The velocity of the particles is color-coded where the user can switch between a rainbow color scale and a heated body color scale (see Fig. 6). Additionally, the user can switch between different blend modes; *alpha blending* and *additive blending*, and different texture types; *particle quads* and *particle stripes*. In alpha blending, the opaque particles occlude each other. Since colors in additive blending add up when covering each other, the depth perception is improved because of different brightness levels depending on the number of stacked particles.

The application supports up to 2 million particles existing simultaneously. Regarding the memory, the VFX Graph requests approx. 260 MB with the quad texture and approx. 138 MB with the stripe texture. GPU and CPU each are working between 100 FPS and 200 FPS. When the garbage collector is activated, the FPS drops below 60 FPS, but the difference is not noticeable. The measurements were taken on a PC with the specifications: Windows 10 x64 operating system, 3.60 GHz AMD Ryzen 5 3600 6-core processor, 16 GB of RAM (1600 MHz), and an AMD Radeon RX 480 graphics card.

### VR exploration

**Fig. 7:**
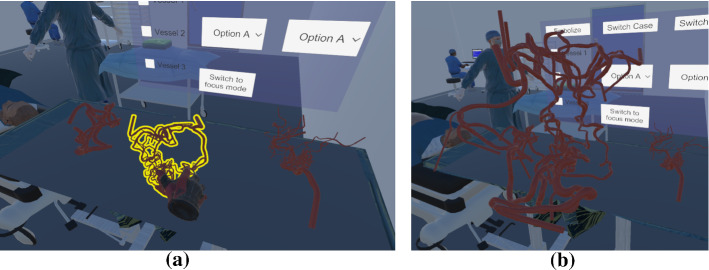
Scene from the application in VR, where the user can select and explore the 3D models of all AVMs

In addition to traditional 2D interaction with mouse and keyboard, we include an immersive VR environment by customizing the virtual surgery room from Huber et al. [12]. Different AVM datasets are placed on a table. The user can pick up individual datasets and explore them. Grabbing is possible with both controllers to rotate and inspect the data (see Fig. 7). This enables a more intuitive and faster exploration of the cases as in the desktop version. The adjustment of parameters is provided by a 2D interface in the virtual environment. For a more detailed exploration, the user can switch to a *focus mode* where she or he is able to fly around the selected case. This method gives the possibility to have a larger model of the AVM. Thus, the complex and small twists of the vessels can be explored more easily. As VR headset, the HTC Vive and its wand controllers were used.

**Fig. 8:**
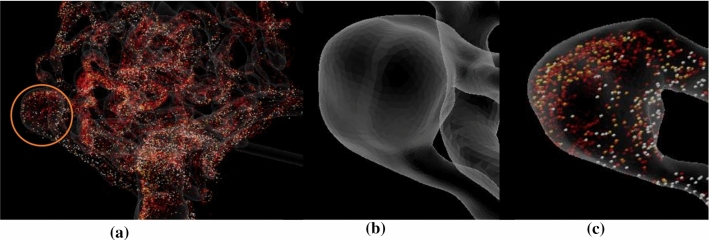
**a** Case 2 with marked aneurysm, **b** focus on 3D model of the marked aneurysm **c** exploring blood flow of the marked aneurysm

**Fig. 9:**
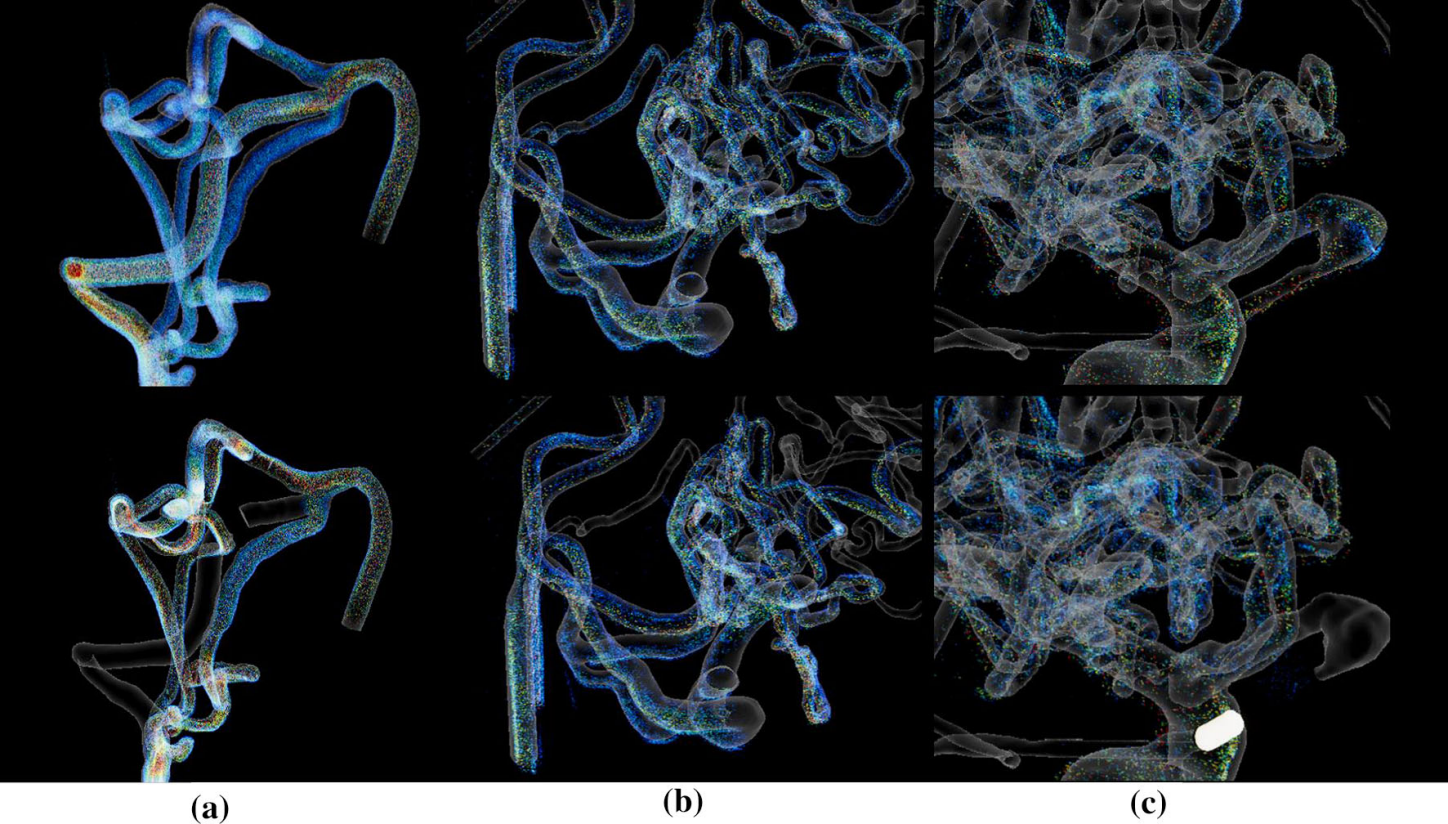
(**a**, top) Case 1 untreated, (**a**, bottom) Case 1 where the main blood feeding artery is embolized, (**b**, top) nidus of Case 2 untreated, (**b**, bottom) Case 2 with six from eight closed feeders, (**c**, top) nidus of Case 3 untreated and (**c**, bottom) Case 3 where the first feeder, which is providing the aneurysm, is closed (embolization is marked with a glowing cylinder)

## Evaluation

In order to evaluate the interactive embolization, we presented the setup, the GUI and the functionalities to two neuroradiologists. One has comprehensive work experience (10 years) regarding interventional treatment of AVMs, whereas the second one has been treating AVMs for more than a year. We used the simplified Case 1 to demonstrate the influence of blocking different feeder arteries. For the evaluation, we used the think-aloud method [20], where the user is encouraged to comment and to provide feedback.

Next, Case 2 was discussed. Both experts were getting familiar with the visualization and controls. Both opted for the heated body color map due to the similarity to reddish blood flow. They also stated that the reconstruction at their angiographic suite usually provides red-colored 3D illustrations. Although the more experienced radiologist favored the additive blending, since increased blood flow and thus the main blood flow direction was visualized by increased brightness due to overlaying of the particles, the novice neuroradiologist stated an interest in slow flow. Regarding the controlling of our application, both favored a control via mouse only instead of our combination of mouse and keyboard.

Both experts pointed out that a virtual treatment of the AVM with a flow-induced aneurysm is highly beneficial (see Fig. 8). Here, they discussed different strategies: blocking the feeder at the aneurysm and evaluating the flow changes within the nidus, or blocking other feeders and evaluating the flow changes in the aneurysm. Here, our application could also serve as a basis for a discussion amongst the physicians working out different treatment strategies. The experienced neuroradiologist pointed out that treatment of flow-induced aneurysms in the presence of an AVM often involves complete blockage of the aneurysm’s parent vessel in order to hamper the blood supply of the AVM which is nicely reflected by our prototype.

The AVM of Case 3 exhibits a comparatively high number of eight feeders and the medical experts stated that for such cases the order of the feeder embolization is crucial in order to avoid large changes in blood pressure. They favor a treatment that supports hemodynamic balance. Then, a possible embolization based on the visual presentation and the virtual embolization was discussed. Thereby, proximally located feeders with small vessel diameters were used as a starting point for the virtual embolization. The feeders with larger vessel diameter and more distal location were then embolized consecutively. This order of embolization serves to maintain the hemodynamic balance of the AVM. Because of the lack of tributary flow into the venous vasculature, which could not be visualized based on the available 3D DSA data, six of the eight existing feeders were closed to still allow flow into the venous system and thus assess the effect of therapy (see Fig. 9).

For a possible usage in the clinical research or practice, both experts requested more quantitative information, like wall shear stress and volume of the blood flow velocity in the nidus related to the parent vessel, as shown for the characterization of intracranial aneurysm blood flow [7]. The novice physician also pointed out that the thickness of vessels could be color-coded as well as their curvature. This is interesting since a catheter-based intervention yields a deformation of the vessels. Instable vessels with strong curvature are prone to rupture and special care must be applied for these. The neuroradiologists also expressed that our application alone is not sufficient to base endovascular treatment on, but it would be a helpful addition for treatment planning in combination with the patient’s medical history, clinical aspects like location and access path planning w.r.t. possible surgery.

## Discussion and limitation

The goal of our work was an embolization simulation without hurting the patient. In general, the blood flow visualization combined with the ghosted view technique to show underlying particles was perceived very well. This design decision is based on previous studies which showed the benefit of these techniques for the presented applications [3, 8]. Based on the evaluation, we identified the following improvements. First, a flexible color scale based on the heated body scale should be provided in both ways: highlighted fast flow as well as slow flow. Second, the application should rely on mouse control only. Third, quantitative parameters like vessel wall thickness, wall shear stresses and reduction in blood flow based on comparisons with the parental artery blood flow would be highly desirable.

The presented work is a pilot study and has several limitations. First, we only use a limited number of datasets since this pathology is rare. Second, controlling via mouse should only be realized instead of combination of mouse and keyboard. Third, regarding the usage of our study for therapy itself and the transferability of the virtually derived embolization order, the clinicians stated that the identification strongly depends on an adequate planning and requires a correct mapping of the 3D model onto the projection views during therapy. Furthermore, one medical expert stated that he would need an overlay of the instrument (i.e., the catheter) for an identification. This would be a nice task for future work since we could include the instrument into the 3D model and extend the presented approach based on forward projections. Then, we could virtually provide 2D projections to support the transfer from virtual treatment planning to therapy. Finally, for the underlying hemodynamic simulations, several assumptions were made. These mainly include rigid wall conditions and a steady flow behavior. Nevertheless, extensive validation studies were carried out in advance  [6, 11, 16, 19] and recommendations regarding the hemodynamic modeling were respected [5]. Hence, plausible flow fields were generated as a proof of concept, which can be extended to pulsatile blood flow in the future.

## Summary

We presented an interactive blood flow visualization of cerebral AVMs in a desktop and immersive virtual reality environment. The user can virtually embolize the AVM feeder arteries and analyze the altered blood flow for a virtual treatment planning. Since our implementation integrates Unity’s VFX Graph, which exploits the graphics card, millions of particles are rendered such that flow patterns can be visualized even in small vessels. We evaluated our approach with two neuroradiologists, who rated the virtual possibility of closing feeder arteries without harming the patient as beneficial for planning of AVM treatment. Although additional clinical information has to be integrated in the planning, our application serves as a pilot study and provides an important supplementation and new possibilities regarding the simulated blood flow analysis.

For future work, the visual exploration application can be extended by quantitative blood flow information listing pressure drops and further hemodynamic parameters.

## Supplementary Information

Below is the link to the electronic supplementary material.

Supplementary material 1 (mp4 37440 KB)
